# Genome-wide association study between CNVs and milk production traits in Valle del Belice sheep

**DOI:** 10.1371/journal.pone.0215204

**Published:** 2019-04-23

**Authors:** Rosalia Di Gerlando, Anna Maria Sutera, Salvatore Mastrangelo, Marco Tolone, Baldassare Portolano, Gianluca Sottile, Alessandro Bagnato, Maria Giuseppina Strillacci, Maria Teresa Sardina

**Affiliations:** 1 Università degli Studi di Palermo, Dipartimento di Scienze Agrarie, Alimentari e Forestali, Italy; 2 Università degli Studi di Palermo, Dipartimento di Scienze Economiche, Aziendali e Statistiche, Italy; 3 Università degli Studi di Milano, Dipartimento di Medicina Veterinaria, Italy; Universita degli Studi di Bologna, ITALY

## Abstract

Copy number variation (CNV) is a major source of genomic structural variation. The aim of this study was to detect genomic CNV regions (CNVR) in Valle del Belice dairy sheep population and to identify those affecting milk production traits. The GO analysis identified possible candidate genes and pathways related to the selected traits. We identified CNVs in 416 individuals genotyped using the Illumina OvineSNP50 BeadChip array. The CNV association using a correlation-trend test model was examined with the Golden Helix SVS 8.7.0 tool. Significant CNVs were detected when their adjusted p-value was <0.01 after false discovery rate (FDR) correction. We identified 7,208 CNVs, which gave 365 CNVRs after aggregating overlapping CNVs. Thirty-one CNVRs were significantly associated with one or more traits included in the analysis. All CNVRs, except those on OAR19, overlapped with quantitative trait loci (QTL), even if they were not directly related to the traits of interest. A total of 222 genes were annotated within the significantly associated CNVRs, most of which played important roles in biological processes related to milk production and health-related traits. Identification of the genes in the CNVRs associated with the studied traits will provide the basis for further investigation of their role in the metabolic pathways related to milk production and health traits.

## Introduction

The availability of several forms of DNA variants, such as single nucleotide polymorphisms (SNPs) and copy number variants (CNVs), has played an important role in phenotypic variation studies. Most genetic and genome-wide association studies (GWAS) have investigated the associations between SNPs as genetic variants and complex and economically important traits, with the aim of identifying subsets of markers able to explain traits [1–5]. CNVs are polymorphic genomic regions, including deletions, duplications and insertions that involve DNA segment ranging from 1 kb to several Mb, that vary compared to a reference genome [6]. CNVs have been shown to be associated with complex traits in several species, including chimpanzees [7], rats [8], and mice [9], and in livestock species such as cattle [10–14], goats [15], and pigs [16, 17]. Like SNPs, these genomic structural variations are considered as important genetic markers of phenotypic variation for complex traits. CNVs have recently been used as markers of phenotypic variation, environmental adaptability, and for economically important traits or disease susceptibility in livestock species [18–20]. However, few studies on CNVs have been published for sheep. Some previous studies [21–23] analyzed CNVs based on comparative genome hybridization arrays, while others [24–27] detected CNVs using SNP microarrays. GWAS using CNVs and phenotypes have been developed in cattle breeds [19, 20, 28–31] and in swine [32, 33]. However, to the best of our knowledge, no GWAS between CNVs detected using the OvineSNP50K BeadChip array and economically important traits (milk, meat, etc) in sheep breeds have been published.

In the current study, we carried out for the first time a GWAS between CNVs and milk-production traits in sheep, with the aim of detecting genomic regions including CNVs affecting these traits.

## Methods

### Ethics statement

Blood samples were collected from sheep by trained veterinarians. All the procedures were approved by the Organismo Preposto al Benessere Animale of the University of Palermo, in agreement with the recommendations of European Union Directive 2010/63/EU, to ensure appropriate animal care.

### Sampling and genotyping

A total of 468 of Valle del Belice sheep from four flocks in Agrigento province (Sicily) were used in this study. About 10 mL of blood was collected from the jugular vein using vacutainer tubes containing EDTA as anticoagulant. Genomic DNA was extracted from blood samples using a salting-out method [34]. DNA amounts for each sample were quantified with NanoDrop ND-1000 spectrophotometer (NanoDrop Technologies, Wilmington, DE, USA), diluted to a final concentration of 50 ng/μl (as required by the Illumina Infinium protocol), and stored at 4°C until use. Genotyping was performed using the Illumina OvineSNP50K BeadChip v2 array containing 54,241 SNPs. The positions of the SNPs on the chromosomes were determined from the ovine Oar_v3.1 genome assembly. All the 468 genotyped individuals passed the quality control criteria of call rate > 98%.

#### Quality control of CNVs

CNVs were detected using Golden Helix SNP & Variation Suite (SVS) 8.7.0 software (Golden Helix, Inc., Bozeman, MT, USA; www.goldenhelix.com). We imported the Log R Ratio (LRR) values for each SNP from GenomeStudio 2.0 software (Illumina Inc.) into SVS.

Unmapped SNPs and sex chromosomes were excluded from the analysis, leaving 52,413 markers for CNV mapping.

Quality assurance of the LRR data and filtering of outlier samples were performed using SVS software, as described by Pinto et al. [35]. The derivate spread log ratio analysis was first used to identify individuals with low-quality LRRs and samples showing genomic waves of LRR signal intensity were subsequently discarded using the genomic wave detection and correction algorithm [36]. Principal component analysis (PCA) was applied to detect and correct for the presence of batch effects and to correct the LRR values. Fifty-two samples were excluded and the remaining 416 were used for CNV detection. All data from GenomeStudio 2.0 software (Illumina Inc.) related to Log R Ratio (LRR) and B allele frequency (BAF) for all individuals are provided in the S1 Dataset.

#### CNV and CNVR detection

The optimal segmenting (CNAM) module of SVS 8.7.0 was used to identify CNVs using the univariate approach that segments each sample independently [20]. The following options in CNAM were used: univariate outlier removal; maximum number of 100 segments per 10,000 markers; minimum markers per segment 3; 2,000 permutations per pair with a p-value cutoff of 0.005. Individuals with a waviness factor (WF) −0.05>WF>0.05 were also excluded, as suggested by Diskin et al. [36]. CNVRs were determined by aggregating overlapping CNVs identified in two or more samples as reported by Redon et al. [37].

#### Phenotypic data

A total of 5,586 lactation records were collected for 481 Valle del Belice sheep from four different flocks between 2009 and 2015. The following phenotypic data were recorded according to a monthly Test Day (TD) scheme: daily milk yield (MY), milk fat percentage (FP), fat yield (FY), protein percentage (PP), protein yield (PY), and milk somatic cell count (SCC). SCC was log-transformed to give the somatic cell score (SCS), using the formula of Ali and Shook [38]. Data were edited using S.A.S. version 9.2 [39] and individuals with fewer than three test-day records or with missing information for any trait were excluded from the dataset. After editing, the observations were reduced to 5,446 phenotypic measures for each trait for each of the 468 individuals (S2 Dataset).

#### Breeding values for milk production traits

A single trait repeatability test-day animal model was used to estimate breeding values (EBVs) for the five milk production traits: MY, FY, FP, PY, and PP, and for SCS using the REMLF90 program [40]. Briefly, the model included fixed (i.e. parity, litter size, season of lambing) and random effects (additive genetic, permanent environmental, and herd by test-day interaction effects). Several models were tested to explore how to fit days in milk and to optimize the analysis. Comparisons between the residuals obtained from the different models showed no significant differences (data not shown). Days in milk were then included as a linear covariate in the model to account for lactation stage as reported by Leitner et al. [41]. The available pedigree information included 5,175 animals including 180 rams and 2,549 dams. Estimated breeding values for all traits were deregressed (DEBVs) according to Garrick et al. [42] as follows:

$$\text{DEBV}=\frac{\text{EBV}}{{\text{r}}^{2}}$$

where EBV is the estimated breeding value of each individual considering each milk production trait and r^2^ is the reliability of that EBV. The DEBVs were considered as more accurate estimates of expected phenotypes and were used as response variables in the GWAS analysis. The descriptive statistics for the DEBVs of each trait are summarized in Table 1.

**Table 1:** Descriptive statistics for DEBVs of the productive and the health traits.

Trait	Individuals	Mean	Standard Deviation	Min	Max
MY	468	100.66	273.40	-797.92	1412.61
FP	468	0.01	0.04	-0.16	0.16
FY	468	7.90	17.88	-5163	90.38
PP	468	0.02	0.04	-0.14	0.18
PY	468	7.29	15.48	-79.54	77.98
SCS	468	0.001	418.20	-44.29	0.01

#### Genome-wide association study

A total of 416 individuals with both CNVs and DEBVs were applied to the GWAS. Association tests were carried out using the Correlation-trend test’ plugin in SVS 8.7.0 software with PCA correction. Significant CNVs were detected when their adjusted p-value was <0.01 after FDR correction. Only CNVRs from significantly associated CNVs with at least one trait were considered for the annotation step.

#### Validation of association test

A simulation study to identify/confirm those CNV calls resulted significantly associated with phenotypes of interest was performed by R software 3.5.1 [43]. In order to test the association for each investigated trait, the 70% of observations was randomly sampled from the whole dataset to build a reference dataset. The remaining 30% of observations are used as independent validation dataset useful to identify/confirm the associations or at least the direction of effects. Moreover, for each replicate only those CNVs resulted associated in the reference dataset were used. This splitting procedure was replicated one hundred times and the association was validated if the CNV was associated both in the reference and the validation dataset at least one time.

#### Gene contents and functional annotation

The gene content of the CNVRs was assessed using *Ovis aries* v3.1 in the Genome Data Viewer genome browser (https://www.ncbi.nlm.nih.gov/genome/gdv/browser/?context=gene&acc=101104604).

Moreover, we performed an enrichment analysis using the Sheep Quantitative Trait Locus (QTL) Database (https://www.animalgenome.org/cgi-bin/QTLdb/OA/index) to identify CNVRs that overlapped QTL regions (QTLRs). We removed the QTLRs that were >5 Mb, and only considered those overlapping at least 50% of each CNVR.

We investigated gene function using the DAVID Bioinformatics Resources 6.8 (https://david.ncifcrf.gov/summary.jsp) for Gene Ontology (GO) analysis and the Kyoto Encyclopedia of Genes and Genomes (KEGG) database (http://www.genome.jp/kegg/pathway.html) for pathway analysis.

## Results and discussion

This study investigated a GWAS between CNVs and milk-production traits in sheep. Although this is the first time that a similar study was performed on sheep, several genomic regions including CNVs affecting these traits have been identified. However, considering the relative low number of individuals affecting the power of the association analysis, our results constitute a preliminary report on the association between these markers and quantitative traits in sheep. Therefore, further analysi on a wider sample could provide more robust results and could be of value for future studies.

### CNV and CNVR detection

CNAM univariate segmentation of the SVS 8.7.0 with PCA detected 7,208 CNVs (S1 Table) with an average of 17.32 per sample and an average length and median size of 348.1 kb and 231.25 kb, respectively. After aggregating the overlapping CNVs, a total of 365 CNVRs were identified (S2 Table) covering 118.36 Mb, corresponding to 4.8% of the genomic sequence of the autosomes and 4.05% of the total genome length. The average number of CNVs involved in CNVRs was 14.58 per individual, with an average length and median size of 324.27 kb and 172.58 kb, respectively. This is partially due to the fact that the BeadChip SNP50K assay was originally developed for high-throughput SNP genotyping in association studies. Then, the length of the identified CNVRs might be larger due to low density and non-uniform distribution of SNPs in Bead Chip SNP50K as reported by Hou et al. [14]. Forty-three CNVRs only included gains (duplications) and 320 only included losses (deletions). As reported by Prinsen et al. [44] and Liu et al. [24], it is expected that SVS tool detected more losses than gains. This is due to the higher power in methodology to identify homozygous losses (where both copies of a locus are lost) linearly correlated to exponential intensity signals. Similar results in terms of coverage (141.6 Mb and 5.8% of genomic sequence of autosomes) were obtained by Yan et al. [27] using the Illumina OvineSNP50K BeadChip with SVS software. Moreover, the same authors [27] demonstrated the characteristics of the CNVRs depended on the algorithm used (SVS, PennCNV, and cnvPartition) in terms of the number of detected CNVRs, total length, and percentage of coverage of the autosomal genome. Differences in the array density and software/algorithm used may thus mean that our results were not totally comparable with other studies on sheep [23–26] and cattle [11–14].

### CNV association analyses

**Fig 1:**
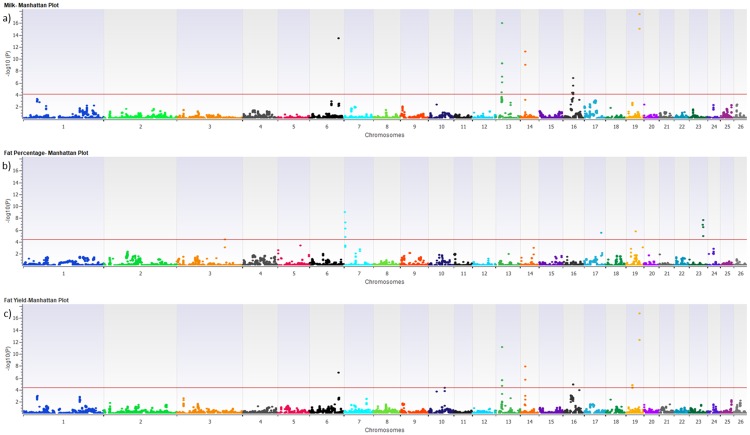
Manhattan plots of–Log10 (p-*value*) for milk yield (a), fat percentage (b) and fat yield (c).

**Fig 2:**
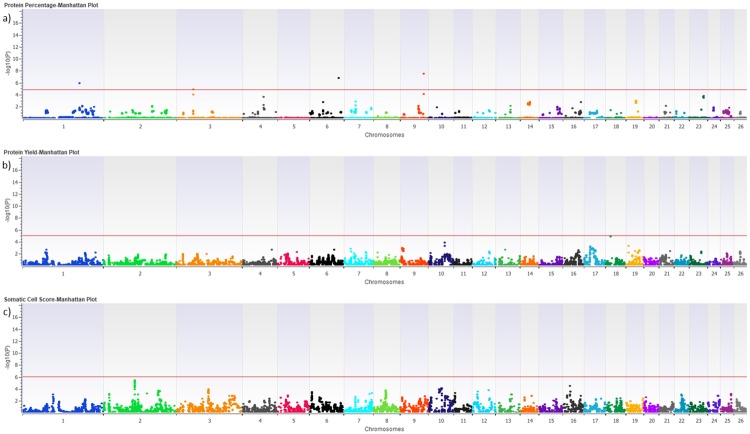
Manhattan plots of–Log10 (p-*value*) for protein percentage (a), protein yield (b) and somatic cell score (c).

Association analysis for each trait was performed for 416 individuals with both CNV and DEBV information. After the CNVs merge, the results showed 31 significant CNVRs associated with the studied phenotypic traits (FDR corrected p-value<0.01) (Table 2). The results of the CNV-association analyses for milk production and the SCS traits are shown as Manhattan plots in Figs 1 and 2. In the Manhattan plots, the 0.01 FDR significance thresholds were shown with red colored line. Regarding the production traits, 13, 11, 10, and two CNVRs were significantly associated with MY, FP, FY, and PP, respectively. No CNVR was significantly associated with PY and SCS, and there is therefore no threshold line in the PY and SCS plots. Of the 31 associated CNVRs, only CNVR_1 identified on chromosome 6 was simultaneously associated with three production traits (MY, FY, PP), three CNVRs on chromosomes 13, 14, and 19 were associated with two traits, while the remaining CNVRs (27) were each associated with only one trait (Table 2).

**Table 2:** List of CNVRs significantly associated with MY, FP, FY and PP traits.

N°	Chr	Start	End	Length	IND	Genes	MY	FP	FY	PP	QTL
CNVR_1	6	98915609	99081950	166342	2	CDS1	6.748E-11		0.000229	0.0006981	QTL:14011 Milk
CNVR_2	7	3206482	3429935	223454	4			7.99E-06			
CNVR_3	7	18487054	18787472	300419	2	MYO9A, LOC105615633, LOC105615632, SENP8, GRAMD2, LOC101110145		0.0008319			
CNVR_4	7	22026509	22172957	146449	2	LOC101106264, LOC101106528, LOC105613077, LOC105613076, LOC105613075		0.0008319			
CNVR_5	9	78451353	78529564	78212	7					0.0002436	QTL:16015 Milk Fat Yield
CNVR_6	13	19401523	20051589	650067	2	MALRD1	6.051E-07				
CNVR_7	13	20379081	20547863	168783	2	PLXDC2, LOC105616667	6.051E-07		1.89E-08		
CNVR_8	13	32965686	33058560	92875	2	ZEB1			1.89E-08		QTL:57750 Milk Protein
CNVR_9	13	48832966	49706045	873080	27	LOC101117953, LOC101118207, LOC101110166	6.051E-07				
CNVR_10	13	49006951	49706045	699095	41	LOC101117953, LOC101118207, LOC101110166	6.051E-07				
CNVR_11	13	62376369	62539468	163100	2	LOC105606911, CBFA2T2, LOC101117009	8.93E-05				
CNVR_12	14	14964181	15944658	980478	66	NETO2, TRNAM-CAU, ITFG1, LOC105613342, PHKB, LOC101110611, LOC105613638, LOC105602056, LOC105602057, LOC105608343	9.693E-09				QTL:57692 Milk Fat Yield
CNVR_13	14	14964181	15916620	952440	5	NETO2, TRNAM-CAU, ITFG1, LOC105613342, PHKB, LOC101110611, LOC105613638, LOC105602056, LOC105602057, LOC105608343	9.693E-09		2.57E-05		QTL:57692 Milk Fat Yield
CNVR_14	14	15008939	15700496	691558	13	NETO2, TRNAM-CAU, ITFG1, LOC105613342, PHKB, LOC101110611, LOC105613638, LOC105602056, LOC105602057, LOC105608343	9.693E-09				QTL:57692 Milk Fat Yield
CNVR_15	14	17982653	18259322	276670	11	HEATR3, PAPD5, LOC105616847, ADCY7, BRD7	9.693E-09				
CNVR_16	14	43271417	43445098	173682	3	CHST8			2.57E-05		QTL:160872 Somatic Cell Score
CNVR_17	14	44947626	46765514	1817889	2	FXYD3, LGI4, FXYD1, FXYD7, FXYD5, LOC101123657, LOC101102084, LSR, USF2, HAMP, MAG, LOC105607576, LOC105602063, CD22, FFAR1, FFAR3, LOC101103344, FFAR2, LOC105607577, KRTDAP, DMKN, SBSN, GAPDHS, TMEM147, ATP4A, LOC101108333, LOC105607579, LOC105607578, LOC101104171, HAUS5, RBM42, ETV2, LOC101121538, TRNAT-UGU, UPK1A, ZBTB32, KMT2B, IGFLR1, U2AF1L4, PSENEN, LIN37, HSPB6, PROSER3, LOC101122717, ARHGAP33, PRODH2, NPHS1, KIRREL2, APLP1, NFKBID, HCST, TYROBP, LRFN3, SDHAF1, SYNE4, ALKBH6, CLIP3, THAP8, WDR62, OVOL3, POLR2I, TBCB, CAPNS1, LOC101121285			2.57E-05		
CNVR_18	14	45934555	46765514	830960	2	ZNF565, ZNF146, ZNF567, TRNAW-CCA, ZNF461, ZNF382, LOC105616880, ZNF529, ZNF260, LOC105616879, ZNF566, LOC101102665, LOC105616881, ZFP14, LOC101106873, ZNF568, ZNF829, LOC105607590, ZNF793, LOC101122971, LOC105607591, ZNF383, LOC101105680, LOC105607592, LOC101107890, ZNF527, ZNF569, TRNAE-UUC, LOC105607593, LOC105616882, ZNF570, ZNF420, LOC105616883, LOC101108155, LOC101108415, LOC101108680, ZNF790, LOC101102169			2.57E-05		
CNVR_19	16	41463528	41637426	173899	2	PDZD2, LOC105602599	0.0001281				
CNVR_20	17	57986988	58424553	437566	4	LOC105602890, LOC105602891, LOC105602892, MED13L		0.0029188			QTL:57700 Milk Fat Yield, QTL:14002 Somatic Cell Score
CNVR_21	19	16600046	17485391	885346	15	IRAK2, VHL, LOC105603408, BRK1, FANCD2OS, FANCD2, EMC3, LOC105603409, PRRT3, CRELD1, IL17RC, IL17RE, JAGN1, CIDEC, RPUSD3, LOC101103096, LOC105603411, LOC105603410, ARPC4, TADA3, OGG1, CAMK1, BRPF1, CPNE9, MTMR14, LOC105603412, LHFPL4, SETD5, LOC105603622, LOC105603415, THUMPD3, LOC105603414, LOC105603416, LOC105603417, SRGAP3		0.0020466			
CNVR_22	19	33480446	34472912	992467	25	LOC105603452, LOC105603453, SUCLG2, LOC105603455, LOC105603454, LOC105603458, LOC105603456, KBTBD8		0.0020466			
CNVR_23	19	40179873	41112452	932580	41	FHIT, LOC105603489	2.491E-14				
CNVR_24	19	43721683	43797484	75802	8	DNAH12, LOC105603505, TRNAC-GCA, LOC105603504			1.48E-13		
CNVR_25	19	44994104	45113902	119799	7	ERC2			2.05E-09		
CNVR_26	19	46527885	46544501	16617	3	CACNA2D3	2.491E-14		1.48E-13		
CNVR_27	23	12624271	13131785	507515	131	LOC101104705, SYT4		9.671E-05			
CNVR_28	23	12649375	13131785	482411	22	LOC101104705, SYT4		9.671E-05			
CNVR_29	23	37464041	38263780	799740	2	EMILIN2, LPIN2, MYOM1, LOC105604463, MRCL3, LOC101105123, LOC105604464, LOC105604465, TGIF1, DLGAP1, LOC105604469, LOC105604468, LOC105604467, LOC105604466		9.671E-05			
CNVR_30	23	40785469	40920221	134753	57	PTPRM, LOC105604482		9.671E-05			
CNVR_31	23	47820944	48653824	832881	11	ZBTB7C, LOC105604508, LOC105604510, LOC105604509, CTIF, LOC105604511, TRNAS-GGA		9.671E-05			QTL:13906 Milk Yield, QTL:13907 Milk Fat Yield

N°: number of CNVR

Chr: number of chromosomes

Start: start position of CNVR in bp

End: end position of CNVR in bp

Length: length of the CNVR in bp

IND: number of individuals carrying theper CNVR

Genes: genes symbols identified within of the CNVRs referred to NCBI (www.ncbi.nlm.nih.gov)

MY: milk yield, FP: milk fat percentage, FY: fat yield, PP: protein percentage; phenotypic traits with p-value<0.01 after FDR corrected

QTL: Quantitative Trait Locus overlapped with CNVRs referred to Sheep QTL database (https://www.animalgenome.org/cgi-bin/QTLdb/OA/index)

### Association test validation

As expected, PY and SCS traits didn’t show any CNVs associated neither in the original whole dataset nor in the simulated ones.

With regards to MY, all CNVRs associated in the original dataset showed a probability greater than 67% of being associated in the reference dataset and only 6 CNVRs out of 13, i.e., CNVR_1, CNVR_6, CNVR_7 and CNVR_12, CNVR_19; CNVR_26 confirmed the association in the validation dataset at least one time.

For FY, FP, and PP traits, the CNVRs associated in the original dataset showed a probability greater than 74%, 49%, and 57%, respectively, of being associated in the reference one. Moreover, for these traits, 6 CNVRs out of 10 for FY (i.e. CNVR_1, CNVR_7, CNVR_13, CNVR_16, CNVR_25, CNVR_26), 4 CNVRs out of 11 for FP (i.e. CNVR_20, CNVR_22, CNVR_29, CNVR_31), and one out of two for PP (i.e. CNVR_5) confirmed the association in the validation dataset. Nevertheless, the results of the simulation are partially satisfying but the results of those CNVs associated in both reference and validation datasets probably highlight CNV calls useful for further studies.

### Gene content and functional annotations

We investigated the content of the 31 CNVRs showing significant associations with the studies traits in sheep QTL by interrogating the Animal QTL database. All CNVRs, except those on OAR19, overlapped with QTLs, though not directly related to the traits of interest.

A total of 222 genes were annotated within the CNVRs significantly associated with the analyzed traits. However, not all CNVRs contained annotated genes, and two CNVRs did not overlap with any annotated gene. Similar results were reported in previous studies on cattle [30, 31]. This may be due to the genome assembly version, especially in non-human species [31]. Several studies have highlighted the genome-wide distribution of CNVs in regions covering non-coding sequences, thus affecting the regulation of distant target genes [45]. We identified several genes that played important roles in biological processes related to the considered milk traits. *CIDEC*, *LPIN2*, *CDS1*, FFAR1, FFAR2, and FFAR3 genes within different CNVRs, were involved in lipid metabolism. *CIDEC* (CNVR_21) encodes a member of the cell death-inducing DNA fragmentation factor-like effector family, and its encoded protein promotes lipid droplet formation in adipocytes and may mediate adipocyte apoptosis. The *CIDEC* gene is regulated by insulin and its expression is positively correlated with insulin sensitivity, and mutations in this gene may contribute to insulin resistant diabetes [46–49]. *Lipins* (*LPIN2* gene in CNVR_29) have dual functions in lipid metabolism by serving as a phosphatidate phosphatase and transcriptional co-regulators of gene expression [50]. Mouse studies suggested that *LPIN2* gene functioned during normal adipose tissue development, and may play a role in human triglyceride metabolism. This gene represents a candidate gene for human lipodystrophy, characterized by loss of body fat, fatty liver, hypertriglyceridemia, and insulin resistance. The *CDS1* gene (CNVR_6) is known to regulate phospholipid metabolism along with other genes [51], by encoding an enzyme that regulates the amount of phosphatidylinositol available for signaling by catalyzing the conversion of phosphatidic acid to CDP-diacylglycerol. A group of free fatty acid (FFA) receptors, encoded by genes including FFAR1, FFAR2, and FFAR3 (CNVR_17), previously known as *GPR40*, *GPR43*, and *GPR41*, respectively, are receptors for FFAs and enable FFAs to act as signal molecules [52]. Mielenz [53] reported the presence of these genes in bovine mammary gland epithelial cells and in different ovine adipose tissues. It is important to highlight that the fatty acid content and milk fat profile affect the technological properties and the nutritional value of dairy products. Sicilian sheep dairy products are highly appreciated for their taste and flavor, and dairy production is mainly based on high quality PDO cheese as Vastedda della Valle del Belice and Pecorino Siciliano. Our findings could be applied on future selection scheme in Valle del Belice breed oriented towards milk yield and milk composition (protein and fat content) to maintain competitiveness of these products. The *CAPNS1* gene (CNVR_17) showed significant effects on natural drip loss, lightness and intramuscular fat content in sheep [54].

GO and KEGG analyses showed that the functions of the proteins encoded by these genes included a wide spectrum of biological processes, cellular components, molecular functions, and pathways (Table 3),multiple significant categories (p≤0.01). In particular, GO terms for cellular components were strongly represented by GO:0005622 intracellular (14 genes), molecular functions by GO:0003676 nucleic acid binding (14 genes) and, GO:0046872 metal ion binding (14 genes), and biological processes by GO:0006355 regulation of transcription (11 genes) (Table 3). KEGG showed five genes related to ‘Regulation of actin cytoskeleton’ and four related to ‘Oxytocin signaling pathway’ with enrichment of genes involved in several GO terms was observed. We have not discussed in detail all the genomic regions within CNVRs associated with the studied traits in detail, but have focused on selected genes in highly GO enriched terms with reported associations with several specific traits related to livestock. We therefore summarize the functions of the candidate genes within significant CNVRs below. Some genes identified by DAVID software participated in biological processes that are related to milk traits considered in this study. For example, the following genes located in CNVRs on OAR14 were associated with MY and FY traits: *CLIP3* (CNVR_17), which plays roles in the positive regulation of protein phosphorylation (GO:0001934) and glucose transport (GO:0001934) and in fat cell differentiation (GO:0045444); *ADCY7* (CNVR_15), involved in the positive regulation of cAMP biosynthetic process (GO:0030819) and intracellular signal transduction (GO:0035556) directly related to inositol lipid-mediated signaling (GO:0048017); *APLP1* (CNVR_17) involved in the negative regulation of cAMP biosynthetic process (GO:0030818); *CHST8* gene (CNVR_16) involved in carbohydrate biosynthetic process (GO:0016051); *HCST* gene (CNVR_17) in protein phosphorylation (GO:0006468) and positive regulation of phosphatidylinositol 3-kinase signaling (GO:0014068), and *PHKB* (CNVR_12–14 range) involved in glycogen metabolic process (GO:0005977). Furthermore, *MALRD1* gene (CNVR_6) on OAR13 was associated with MY and FY traits via roles in cholesterol homeostasis (GO:0042632) and negative regulation of bile acid biosynthetic process (GO:0070858), while S*UCLG2* (CNVR_22) in OAR19 is involved in metabolic process (GO:0008152), and *ZBTB7C* (CNVR_31) in OAR23 is involved in the positive regulation of fat cell differentiation (GO:0045600). These sets of genes, associated to milk traits, possess a wide spectrum of molecular function and provide a huge resource for testing hypotheses on the genetic basis of phenotypic variation within our breed.

**Table 3:** The enrichment of GO terms associated with the CNVRs genes (*P*–value < 0.05).

Category	GOTerm	GO Name	Count	P-value
GOTERM_CC_Direct	GO:0005622	**intracellular**	**14**	**6.61E-04**
GOTERM_MF_Direct	GO:0003676	**nucleic acid binding**	**14**	**7.27E-06**
	GO:0046872	**metal ion binding**	**14**	**5.78E-04**
GOTERM_BP_Direct	GO:0006355	**regulation of transcription, DNA-templated**	**11**	**1.04E-04**
	GO:0006366	transcription from RNA polymerase II promoter	3	0.041031
	GO:0050832	defense response to fungus	2	0.057082

## Conclusion

At present, limited knowledge is available on association between CNVs and production traits in sheep. To the best of our knowledge, this is the first GWAS of CNVs and milk production traits in dairy sheep breed. Our results indicate that many CNVRs are associated with one or more milk production traits, and probably contribute to phenotypic variation. In particular, the two most significant CNVRs (p-value = 2.49E-14) located on chromosome 19 and associated with MY will be more investigated.

These findings provide a useful basis for the development of breeding programs. The genes identified in the CNVRs associated with the studied traits may be used for more detailed investigation of their roles in the metabolic pathways related to milk production and health traits in sheep. However, future studies based on a wider sample would be particularly relevant to refine and validate our results.

## Supporting information

S1 Dataset Genotypic dataset from GenomeStudio v2.0 software for 468 individuals of Valle del Belice breed (https://www.animalgenome.org/repository/pub/UPIT2018.0803/). (XLSX)

S2 Dataset DEBVs for 468 individuals of Valle del Belice breed (https://www.animalgenome.org/repository/pub/UPIT2018.0803/). (XLSX)

S1 Table List of identified CNVs. (XLSX)

S2 Table List of CNVRs determined by aggregating overlapping CNVs. (XLSX)
